# Inhalable Hsa‐miR‐30a‐3p Liposomes Attenuate Pulmonary Fibrosis

**DOI:** 10.1002/advs.202405434

**Published:** 2025-03-22

**Authors:** Shuo Liu, Kristen D. Popowski, Christina M. Eckhardt, Weihang Zhang, Junlang Li, Yujia Jing, Dylan Silkstone, Elizabeth Belcher, Megan Cislo, Shiqi Hu, Halle Lutz, Asma Ghodsi, Mengrui Liu, Phuong‐Uyen C. Dinh, Ke Cheng

**Affiliations:** ^1^ Department of Biomedical Engineering Columbia University New York NY 10032 USA; ^2^ Department of Molecular Biomedical Sciences North Carolina State University Raleigh NC 27606 USA; ^3^ Comparative Medicine Institute North Carolina State University Raleigh NC 27606 USA; ^4^ Department of Pulmonary Allergy and Critical Care Medicine Columbia University College of Physicians and Surgeons New York NY 10032 USA; ^5^ Xsome Biotech Inc. Raleigh NC 27606 USA; ^6^ Joint Department of Biomedical Engineering University of North Carolina at Chapel Hill and North Carolina State University Raleigh/Chapel Hill NC 27606 USA; ^7^ Department of Biological Sciences North Carolina State University Raleigh NC 27606 USA; ^8^ Department of Food Bioprocessing and Nutrition Sciences North Carolina State University Raleigh NC 27606 USA

**Keywords:** CNPY2, idiopathic pulmonary fibrosis, inhalation, miR‐30a‐3p

## Abstract

Idiopathic pulmonary fibrosis (IPF) remains an incurable form of interstitial lung disease with sub‐optimal treatments that merely address adverse symptoms or slow fibrotic progression. Here, inhalable hsa‐miR‐30a‐3p‐loaded liposomes (miR‐30a) for the treatment of bleomycin‐induced pulmonary fibrosis in mice are presented. It was previously found that exosomes (Exo) derived from lung spheroid cells are therapeutic in multiple animal models of pulmonary fibrosis and are highly enriched for hsa‐miR‐30a‐3p. The present study investigates this miRNA as a singular factor to treat IPF. Liposomes containing miR‐30a mimic can be delivered to rodents through dry powder inhalation. Inhaled miR‐30a and Exo consistently lead to improved pulmonary function across six consecutive pulmonary function tests and promote de‐differentiation of profibrotic myofibroblasts. The heterogenous composure of Exo also promotes reparative alveolar type I and II cell remodeling and vascular wound healing through broad transforming growth factor‐beta signaling downregulation, while miR‐30a targets myofibroblast de‐differentiation through CNPY2/PERK/DDIT3 signaling. Overall, inhaled miR‐30a represses the epithelial‐mesenchymal transition of myofibroblasts, providing fibrotic attenuation and subsequent improvements in pulmonary function.

## Graphical abstract

The study explores inhalable hsa‐miR‐30a‐3p‐loaded liposomes as a treatment for bleomycin‐induced pulmonary fibrosis in mice. Hsa‐miR‐30a‐3p is electroporated into liposomes and formulated into a dry powder for inhalation. Delivered via dry powder inhalation, it improves pulmonary function, promotes myofibroblast de‐differentiation by targeting CNPY2/PERK/DDIT3 signaling, and effectively suppresses the epithelial‐mesenchymal transition, leading to significant fibrosis attenuation.

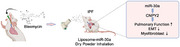

## 1 Introduction

Idiopathic pulmonary fibrosis (IPF) is a chronic form of interstitial lung disease characterized, marked by an imbalance in extracellular matrix (ECM) deposition and altered alveolar histology.^[^ ^1^ ^]^ IPF primarily affects individuals over the age of 65 and has a median overall survival of only 3.8 years.^[^ ^2^ ^]^ IPF is an invariably progressive disease that progresses and worsens as the alveoli sustain repeated micro‐injuries that alter ECM composition and disrupt gas exchange, ultimately leading to impaired lung function and resulting in respiratory failure.^[^ ^1^, ^3^ ^]^ Despite extensive research, the exact etiology of IPF remains unknown, and no current therapies can fully halt or reverse disease progression.

Currently, nintedanib and pirfenidone are the only two antifibrotic drugs approved by the FDA for IPF treatment.^[^ ^4^, ^5^ ^]^ Both drugs have been shown to slow the decline in lung function, as measured by the forced vital capacity (FVC), and delay disease progression.^[^ ^6^, ^7^, ^8^, ^9^ ^]^ However, no available therapies have been shown to reverse lung fibrosis. Further, the clinical efficacy of anti‐fibrotic drugs is often limited by intolerable side effects such as gastrointestinal distress and liver toxicity, which may necessitate discontinuation in many patients.^[^ ^8^, ^9^, ^10^ ^]^ Lung transplantation remains the only curative intervention in IPF, but transplantation is only available for a small number of select patients, and most adults with IPF succumb to progressive respiratory failure. Thus, there is a critical need for novel therapies that can actually reverse interstitial fibrosis while minimizing off‐targeted effects.

Our group has developed lung spheroid cells (LSCs)^[^ ^11^ ^]^ and is currently testing their efficacy in a human clinical trial for patients with IPF (HALT‐IPF, NCT04262167). In preclinical models, LSCs and their secreted products have demonstrated pulmonary regenerative effects in models of both IPF^[^ ^13^, ^14^ ^]^ and coronavirus disease 2019 (COVID‐19).^[^ ^15^, ^16^ ^]^ Notably, extracellular vesicles (EVs) or exosomes (Exo) that are secreted from LSCs are effective delivery vehicles of both exogenous mRNA and protein drugs and are deliverable through nebulization^[^ ^14^, ^15^, ^16^, ^17^ ^]^ and dry powder inhalation (DPI).^[^ ^18^ ^]^ Exosomes are biologically derived nanoparticles (NPs) with known therapeutic effects, depending on the parent cell derivation.^[^ ^19^, ^20^, ^21^ ^]^ Exosomes have gained popularity as therapies and drug delivery vehicles for a variety of diseases,^[^ ^22^, ^23^, ^24^ ^]^ and lung‐derived exosomes may serve as a customizable inhaled therapeutic for respiratory diseases including IPF.^[^ ^17^, ^18^, ^25^ ^]^

Various methods are used for delivering microRNAs (miRNAs), each with its own limitations.^[^ ^26^ ^]^ Intravenous (IV) administration allows systemic distribution but faces challenges including rapid clearance by the livers and kidneys, and off‐target uptake by nontarget tissues.^[^ ^27^ ^]^ Intratracheal instillation ensures high lung concentrations but is more invasive than inhalation methods.^[^ ^28^ ^]^ Intraperitoneal injection bypasses certain circulatory challenges but can result in inconsistent biodistribution.^[^ ^29^ ^]^ Finally, oral delivery faces significant barriers due to enzymatic degradation in the gastrointestinal tract, making it less effective without advanced nanocarrier technologies.^[^ ^27^ ^]^ In contrast, DPI offers a noninvasive method that effectively targets and confines the drug to the lungs, making it a promising approach for delivering miRNA in the treatment of pulmonary fibrosis.

Previously, we identified hsa‐miR‐30a‐3p as a highly enriched miRNA in Exo.^[^ ^14^ ^]^ Several members of the miR‐30 family, including miR‐30a‐3p and miR‐30a‐5p, are significantly decreased in lung tissue^[^ ^30^ ^]^ and bronchoalveolar lavage fluid (BALF) from IPF patients.^[^ ^31^ ^]^ Delivery of miR‐30a‐3p mimics in both human in vitro and rodent in vivo experiments attenuated pro‐fibrotic signaling pathways and highlighted miR‐30a‐3p as a potential therapeutic target for IPF.^[^ ^32^, ^33^ ^]^ Because Exo contains hsa‐miR‐30a‐3p and demonstrates tissue regeneration in bleomycin‐induced IPF, we hypothesized that liposomes containing miR‐30a‐3p mimic can attenuate lung fibrogenesis and improve pulmonary function in animal models of pulmonary fibrosis. To evaluate hsa‐miR‐30a‐3p as a singular factor, we loaded a miR‐30a‐3p mimic into synthetic liposome NPs (miR‐30a) and delivered them through DPI to mice with pulmonary fibrosis. We evaluated tissue remodeling, pulmonary function, and changes in biological signaling pathways in comparison to mice treated with Exo via DPI.

## 2 Results

### 2.1 Characterization of Inhaled Nanoparticles

**Figure 1:**
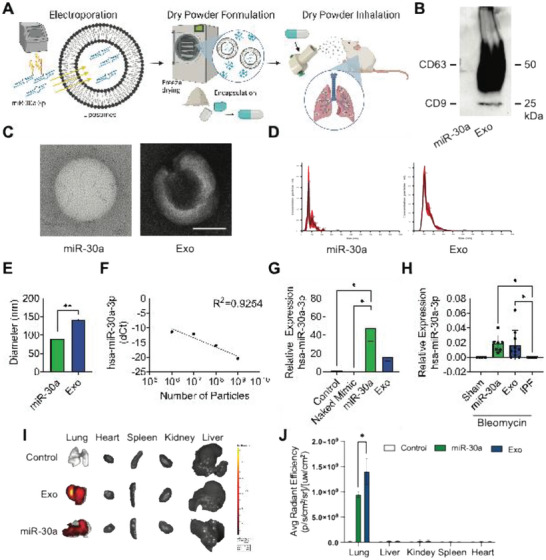
Characterization of nanoparticles. A) Schematic of hsa‐miR‐30a‐3p loading into liposomes, dry powder formulation, and dry powder inhalation. Created with BioRender.com. B) Immunoblots of CD63 and CD9 in NP lysate. C) TEM images of miR‐30a and Exo; scale bar = 50 nm. D) NTA size distribution analysis of NPs. E) Quantification of NTA size distribution analysis of the average mean ± standard error of three replicates. F) Standard curves of the normalized expression (dCt) of hsa‐miR‐30a‐3p to the LSC Control in LSC RNA co‐cultured with miR‐30a Lipo by qPCR; *n* = 2 per particle number. G) Relative expression (ddCt) of hsa‐miR‐30a‐3p to the RNU6 control gene and LSC Control in LSC RNA co‐cultured with Naked Mimic, miR‐30a, or Exo by qPCR; *n* = 2 per group. H) Relative expression (ddCt) of hsa‐miR‐30a‐3p to the RNU6 control gene and Sham in murine lung RNA that received DPI treatments of miR‐30a, Exo, or PBS (Sham and IPF) by qPCR; *n* = 4–10 per group. I,J), Representative ex vivo images I) and quantitative analysis J) of mouse major organs that received miR‐30a and Exo before and 24 h post‐inhalation. The control group consists of mice that did not receive any treatment. *p* values were determined by one‐way ANOVA using GraphPad PRISM software. ^*^ *p* < 0.05, ^**^ *p* < 0.01, ^***^ *p* < 0.001, ^****^ *p* < 0.0001; *ns*, not significant.

The hsa‐miR‐30a‐3p miRNA mimic was loaded into liposomes via electroporation and lyophilized for dry powder inhalation delivery (**Figure** **1**A). The NPs (miR‐30a and Exo) were characterized by immunoblotting (Figure 1B), transmission electron microscopy (TEM) (Figure 1C), and nanoparticle tracking analysis (NTA) (Figure 1D). Characterization confirmed that Exo contained the exosomal phenotype (CD63^+^ and CD9^+^) while miR‐30a lacked the exosomal phenotype. miRNA loading did not alter membrane integrity and generated NPs that had significantly smaller mean diameters compared to Exo (Figure 1E). To evaluate miRNA loading and dosage, standard curves of cellular hsa‐miR‐30a‐3p expression were generated by quantitative polymerase chain reaction (qPCR) (Figure 1F). LSCs co‐cultured with miR‐30a showed significantly greater relative expression of hsa‐miR‐30a‐3p in comparison to Naked Mimic and the Control (Figure 1G). Next, the delivery of hsa‐miR‐30a‐3p was evaluated in mouse models of bleomycin‐induced pulmonary fibrosis, in which mice received seven consecutive daily doses of miR‐30a or Exo through DPI. Relative expression of hsa‐miR‐30a‐3p from lung RNA revealed mice that received miR‐30a and Exo had significantly greater relative expressions of hsa‐miR‐30a‐3p in comparison to healthy mice that received PBS (Sham) and mice with pulmonary fibrosis that received PBS (IPF) (Figure 1H). Furthermore, we utilized *ex vivo* imaging to assess the distribution of DiD‐labeled miR‐30a and Exo following inhalation. Twenty four hours post‐inhalation, both miR‐30a and Exo were predominantly localized in the lungs, with minimal presence observed in other organs including the heart, spleen, kidney, and liver (Figure 1I,J).

### 2.2 Inhalation of Hsa‐miR‐30a‐3p Improves Pulmonary Function

**Figure 2:**
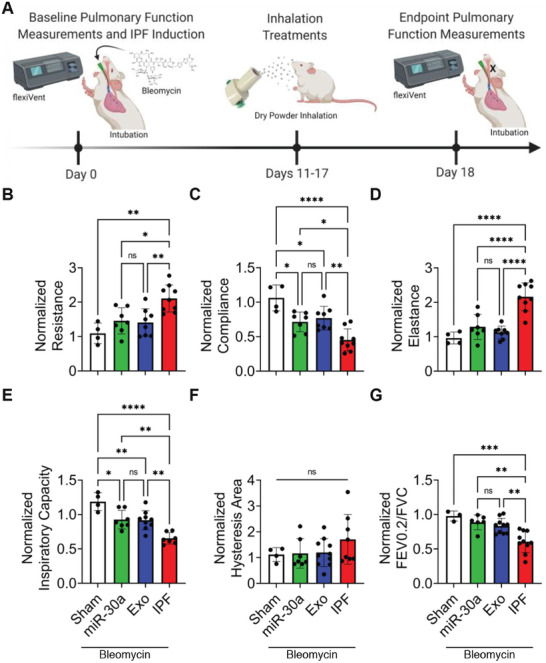
Pulmonary function changes following inhalation of nanoparticles. A) Schematic of baseline pulmonary function measurements, DPI treatments, and endpoint pulmonary function measurements. Created with BioRender.com. B) Quantification of endpoint resistance normalized to baseline resistance per mouse; *n* = 4–10 per group. C) Quantification of endpoint compliance normalized to baseline compliance per mouse; *n* = 3–10 per group. D) Quantification of endpoint elastance normalized to baseline elastance per mouse; *n* = 4–10 per group. E) Quantification of endpoint inspiratory capacity normalized to baseline inspiratory capacity per mouse; *n* = 4–9 per group. F) Quantification of endpoint hysteresis area normalized to baseline hysteresis area per mouse; *n* = 4–10 per group. G) Quantification of endpoint FEV0.2/FVC normalized to baseline FEV0.2/FVC per mouse; *n* = 4–10 per group. *p* values were determined by one‐way ANOVA using GraphPad PRISM software. ^*^ *p* < 0.05, ^**^ *p* < 0.01, ^***^ *p* < 0.001, ^****^ *p* < 0.0001; *ns*, not significant.

Pulmonary fibrosis leads to reduced lung compliance and lung function impairment. Here, healthy mice underwent several pulmonary function tests (PFTs) to establish baseline measures of lung function. Pulmonary fibrosis was then induced through intratracheal instillation of bleomycin and PFTs were reassessed. IPF mice had pulmonary function values consistent with expected clinical findings: increased values in resistance (**Figure** **2**B), elastance (Figure 2D), and hysteresis area (Figure 2F), and decreased values in compliance (Figure 2C), inspiratory capacity (Figure 2E), and forced expiratory volume to FVC (FEV0.2/FVC) (Figure 2G). Further, The bleomycin‐induced IPF model responded effectively to the antifibrotic treatment with Pirfenidone (Figure S3, Supporting Information). Seven consecutive doses of NPs were then delivered via DPI and PFTs were reassessed (Figure 2A). Notably, miR‐30a and Exo treatments demonstrated consistent improvement in measures of pulmonary function (Figure 2B–G). Relative improvements in pulmonary function were similar in miR‐30a and Exo‐treated mice, and pulmonary function values were not significantly different across six consecutive PFTs. These findings were further corroborated by the quantification of serum biomarkers of fibrotic lung remodeling, MMP7 and MUC1, which were reduced after delivery of therapeutic NPs (Figure S4, Supporting Information). Interestingly, inhaled miR‐30a and Exo also improved liver function by reducing serum alanine transaminase (ALT) and aspartate aminotransferase (AST) levels (Figure S1, Supporting Information).

### 2.3 Inhalation of Hsa‐miR‐30a‐3p Induces Anti‐Fibrotic Pulmonary Tissue Remodeling

**Figure 3:**
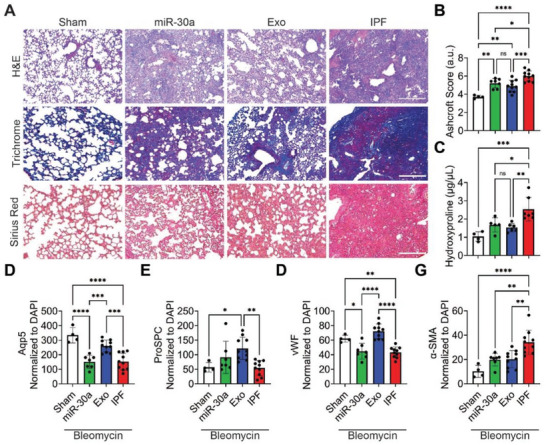
Tissue remodeling and regeneration following inhalation of nanoparticles. A) Representative H&E, Gomori's trichrome, and sirius red images of mouse lung tissue sections after DPI treatments; scale bar = 500 µm for H&E and trichrome sections and 250 µm for sirius red sections. B) Quantification of fibrosis by Ashcroft scoring; *n* = 4–10 per group. C) Quantification of hydroxyproline concentrations from murine lung tissue; *n* = 4–7 per group. D) Quantification of Aqp5 pixel intensity from mouse lung tissue images normalized to nuclei; *n* = 4–10 per group. E) Quantification of ProSPC pixel intensity from mouse lung tissue images normalized to nuclei; *n* = 4–10 per group. F) Quantification of vWF pixel intensity from mouse lung tissue images normalized to nuclei; *n* = 4–10 per group. G) Quantification of α‐SMA pixel intensity from mouse lung tissue images normalized to nuclei; *n* = 4–10 per group. *p* values were determined by one‐way ANOVA using GraphPad PRISM software. ^*^ *p* < 0.05, ^**^ *p* < 0.01, ^***^ *p* < 0.001, ^****^ *p* < 0.0001; *ns*, not significant.

Next, we evaluated pulmonary tissue remodeling after DPI treatments. Histological staining demonstrated a reduction of fibrotic (hematoxylin and eosin (H&E)‐ purple) and collagenous (trichrome‐ blue; Sirius red‐ pink) regions in the lungs of mice that received inhaled miR‐30a and Exo (**Figure** **3**A). Quantification of fibrotic area by Ashcroft Score revealed that both miR‐30a and Exo treatments reduced the measured fibrotic area (Figure 3B). Quantification of total collagen deposition from lung lysate by hydroxyproline concentration (Figure 3C) showed consistent reductions. To evaluate cellular changes following treatments, we used immunohistochemistry analysis to evaluate alveolar type I (ATI) cells by aquaporin 5 (Aqp5), alveolar type II (ATII) cells by prosurfactant protein C (ProSPC), vascular wound healing by von Willebrand factor (vWF), and myofibroblasts by alpha‐smooth muscle actin (α‐SMA) (Figure S2, Supporting Information). Exo treatments facilitated ATI (Figure 3D) and ATII (Figure 3E) proliferation, vascular wound healing (Figure 3F), and de‐differentiation of pulmonary myofibroblasts (Figure 3G). miR‐30a treatments de‐differentiated pulmonary myofibroblasts but could not restore ATI, ATII, or vWF levels.

### 2.4 Inhalation of Hsa‐miR‐30a‐3p Attenuates Pulmonary Fibrosis by Downregulating Epithelial‐Mesenchymal‐Transition

**Figure 4:**
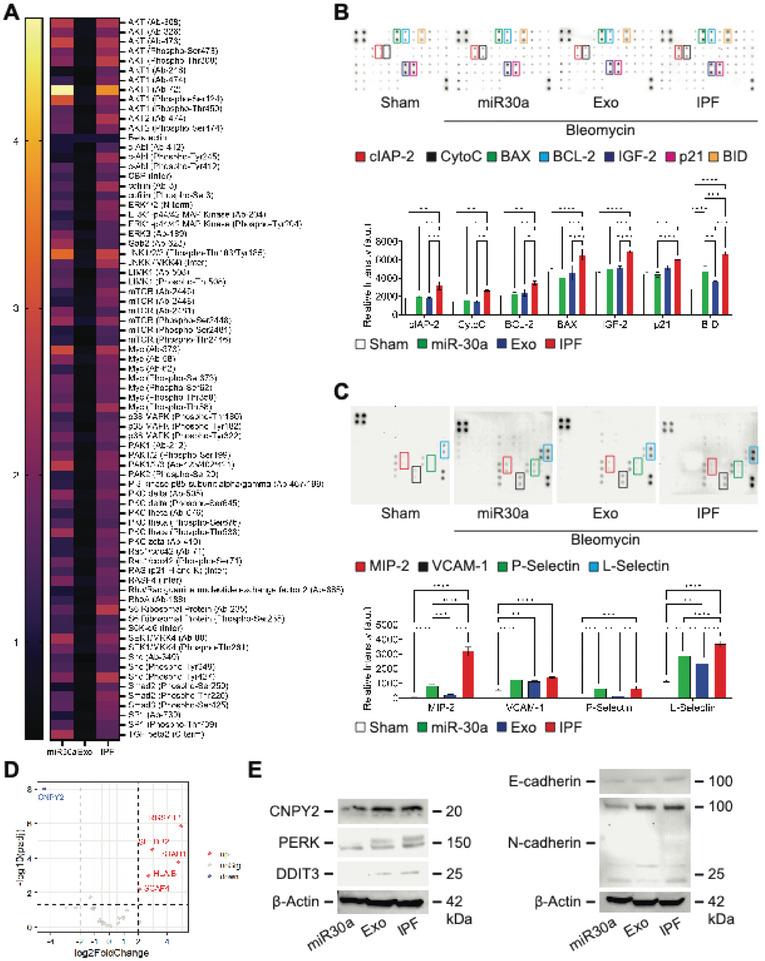
Inhaled hsa‐miR‐30a‐3p downregulates CNPY2/PERK/DDIT3 signaling. A) Heatmap of TGF‐β signaling genes normalized to Sham and β‐actin from lung tissue lysate. Detection of genes is in duplicates. B) Apoptosis array from lung tissue lysate. Detection of genes is in duplicates. C) Cytokine array from mouse serum. Detection of genes is in duplicates. D) Volcano plot of target genes of hsa‐miR‐30a‐3p. Red indicates significantly upregulated genes, grey indicates not significant genes and blue indicates significantly downregulated genes. Detection of genes is in duplicates. E) Immunoblots of CNPY2, PERK, DDIT3, E‐cadherin, N‐cadherin, and β‐actin in lung tissue lysate. *p* values were determined by one‐way ANOVA using GraphPad PRISM software. ^*^ *p* < 0.05, ^**^ *p* < 0.01, ^***^ *p* < 0.001, ^****^ *p* < 0.0001; *ns*, not significant.

To elucidate the mechanisms of miR‐30a and Exo, we examined transforming growth factor‐beta (TGF‐β) and apoptotic signaling pathways from lung tissue lysate, systemic cytokines from serum, and target genes of hsa‐miR‐30a‐3p in lung RNA. It is well known that TGF‐β signaling activation plays a central role in IPF^[^ ^1^ ^]^ and Exo treatments demonstrated broad downregulation of pro‐TGF‐β signaling (**Figure** **4**A). miR‐30a‐ and Exo‐treated mice demonstrated similar reductions in apoptotic markers including cellular inhibitors of apoptosis protein 1 (cIAP‐2), cytochrome c (CytoC), B‐cell lymphoma‐2 (BCL‐2), BCL‐2 associated X‐protein (BAX), insulin‐like growth factor 2 (IGF‐2), cyclin‐dependent kinase inhibitor 1 (p21), and BH3 interacting domain death agonist (BID) (Figure 4B). Systemically, Exo treatments better reduced inflammatory protein 2 (MIP‐2), vascular cell adhesion protein 1 (VCAM‐1), P‐Selectin, and L‐Selectin cytokines compared to miR‐30a treatments (Figure 4C). To identify enrichment for target genes of hsa‐miR‐30a‐3p, we generated volcano plots of fold enrichment (log2FoldChange) of lung RNA from mice treated with miR‐30a versus IPF mice. Of the top target genes of hsa‐miR‐30a‐3p, mice that received miR‐30a showed significant downregulation of canopy fibroblast growth factor signaling regulator 2 (CNPY2) compared to IPF mice (Figure 4D). Mice that received inhaled miR‐30a revealed decreased CNPY2 protein expression, as well as downstream protein kinase RNA‐like endoplasmic reticulum kinase (PERK) and DNA damage‐inducible transcript 3 (DDIT3) expression (Figure 4E). In addition, miR‐30a treatments repressed epithelial‐mesenchymal transition (EMT) by downregulating N‐cadherin and upregulating E‐cadherin (Figure 4E).

### 2.5 Hsa‐miR‐30a‐3p Regulates CNPY2/PERK/DDIT3 Signaling Pathway

**Figure 5:**
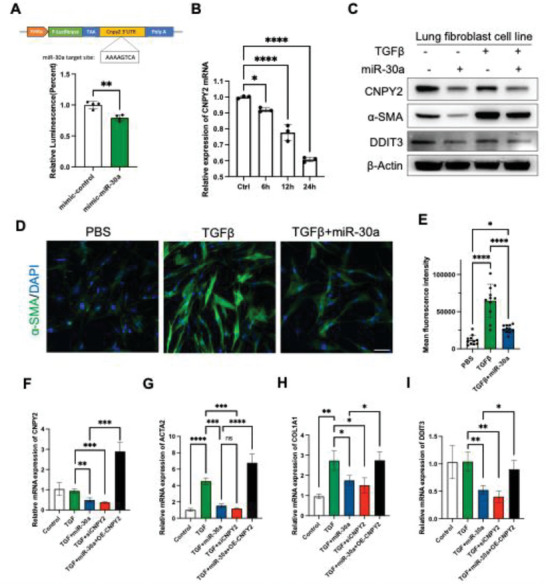
The hsa‐miR‐30a‐3p regulates fibrosis through CNPY2. A) Duel luciferase reporter assay to determine miR‐30a‐CNPY2 mRNA interactions. B) Relative CNPY2 mRNA expression after hsa‐miR‐30a‐3p transfection in lung fibroblast cells. The control group is from cells that did not receive hsa‐miR‐30a‐3p transfection. C) CNPY2, α‐SMA, and DDIT3 expression in lung fibroblast cells after TGFβ and miR‐30a treatment. D) Immunostaining of α‐SMA in TGFβ and hsa‐miR‐30a‐3p treated lung fibroblast cells. Scale bar = 30 µm. E) Quantification of D). F‐I) Relative mRNA expressions after transfections indicated. The control groups are from cells that did not receive any treatment. *n* = 3 per group. *p* values were determined by one‐way ANOVA using GraphPad PRISM software. ^*^ *p* < 0.05, ^**^ *p* < 0.01, ^***^ *p* < 0.001, ^****^ *p* < 0.0001; *ns*, not significant.

Through RNA sequencing, we identified CNPY2 as a potential target of hsa‐miR‐30a‐3p. To further investigate its regulatory role, we inserted the CNPY2 3′UTR, which contains the predicted hsa‐miR‐30a‐3p target site, into the pGL3‐promoter vector, downstream of the TAA sequence and upstream of the polyadenylation signal (**Figure** **5**A, upper). Subsequent dual‐luciferase reporter assays revealed a significant reduction in luminescence in lung fibroblast cells transfected with hsa‐miR‐30a‐3p (Figure 5A, lower). Additionally, we measured CNPY2 mRNA expression levels post‐hsa‐miR‐30a‐3p transfection in lung fibroblast cells, observing a time‐dependent decrease in expression within 24 h (Figure 5B). Protein level assessments also showed a decrease in CNPY2 expression following hsa‐miR‐30a‐3p treatment (Figure 5C). To further validate our results, primary lung fibroblasts from IPF patients were utilized. CNPY2 and α‐SMA expression were notably upregulated in IPF lung fibroblast and reduced after hsa‐miR‐30a‐3p treatment (Figure S5A, Supporting Information). Additionally, E‐cadherin levels in IPF lung fibroblasts were lower compared to controls but were upregulated upon hsa‐miR‐30a‐3p administration, suggesting a regulatory role of hsa‐miR‐30a‐3p in EMT and lung fibrogenesis (Figure S5B, Supporting Information). Collectively, these data support the hypothesis that hsa‐miR‐30a‐3p directly targets the 3′UTR of CNPY2, thereby regulating CNPY2 expression.

α‐SMA is recognized as a fibrosis marker. Its levels are significantly elevated following TGFβ treatment but decrease upon hsa‐miR‐30a‐3p transfection (Figure 5C–E). Additionally, the expression of Ddit3, a downstream target of CNPY2, is also diminished post hsa‐miR‐30a‐3p transfection. This underscores hsa‐miR‐30a‐3p's pivotal role in modulating fibrosis by regulating CNPY2 expression. To further investigate whether hsa‐miR‐30a‐3p's regulatory effect is mediated specifically through CNPY2 and not other genes, we overexpressed CNPY2 in lung fibroblast cells using CNPY2 expression plasmids lacking the 3′ UTR. The expression levels of Acta2, Col1a1, and Ddit3 were significantly decreased following treatment with either miR‐30a or CNPY2 siRNA but were restored upon CNPY2 overexpression (Figure 5F–I). These findings highlight the critical role of hsa‐miR‐30a‐3p in regulating fibrosis through CNPY2 expression modulation.

## 3 Discussion

Current antifibrotic drugs fail to attenuate pulmonary fibrosis and are accompanied by adverse side effects that often necessitate drug discontinuation. Here, we demonstrate the therapeutic efficacy of miR‐30a and Exo as inhaled antifibrotic drugs that reverse interstitial scarring without eliciting pro‐inflammatory responses.

Both miR‐30a and Exo maintained their vesicular integrities and cargo efficacies upon miRNA loading, lyophilization, and DPI delivery. miR‐30a and Exo deliver functional hsa‐miR‐30a‐3p cargo when co‐cultured with LSCs, as well as to rodent lungs through DPI. We have previously reported inhaled delivery of exosomes and liposomes through nebulization^[^ ^19^ ^]^ and DPI,^[^ ^18^ ^]^ and determined that both nanoparticles deliver functional mRNA and protein cargo from the nasal cavity to the deep lung. By utilizing inhaled delivery, drug dosage is optimized by avoiding systemic delivery. In comparison, currently, available small‐molecule drugs including nintedanib and pirfenidone are delivered orally and demonstrate dose limitations and many adverse drug interactions.^[^ ^8^, ^9^ ^]^ With notable alveolar scarring in the bleomycin‐induced IPF mouse model, NPs like miR‐30a and Exo are delivered directly to affected tissue while minimizing systemic exposure.

While current treatments for IPF focus on symptom management or merely slowing disease progression, both miR‐30a and Exo successfully restore lung function across six consecutive measures of lung function. FVC is an established clinical measure of IPF severity and progression and inhaled miR‐30a and Exo both significantly improved FEV0.2/FVC values by the study endpoint. With symptoms such as shortness of breath being common in IPF patients, miR‐30a and Exo can also improve inspiratory capacity, which may increase a patient's quality of life. Current anti‐fibrotic therapies for IPF are often discontinued due to liver toxicity. In contrast, miR‐30a and Exo treatments showed reductions in serum ALT and AST. Because inhaled exosomes and liposomes are metabolized in the liver,^[^ ^17^ ^]^ miR‐30a and IPF may have additional off‐targeted hepatic benefits in IPF.

The miR‐30a and Exo demonstrated robust restoration of pulmonary structures upon histopathological evaluation. Both inhaled NPs significantly reduced the overall fibrotic area as well as collagen deposition in the lungs. While Exo‐ treated lungs showed smaller fibrotic nodules, both miR‐30a and Exo restored the alveolus (H&E) and lung matrix (trichrome and sirius red) closer to a healthy Sham pathology. When evaluating specific cellular changes after inhalation treatments, Exo revealed the regeneration of ATI^+^ and ATII^+^ cells, promoted vascular wound healing, and promoted de‐differentiation of pulmonary myofibroblasts. Exo‐treated lungs also showed a well‐distributed ATI^+^ alveolar epithelium and a proliferation of ATII alveolar stem cells. While miR‐30a treatments could not restore the alveolar epithelium or pulmonary vasculature, the treatment did promote it and showed robust myofibroblast de‐differentiation. Pro‐fibrotic nodules were absent in miR‐30a‐ and Exo‐treated lungs and de‐differentiation of myofibroblasts corresponded with reduced collagen deposition.

Apoptotic and proinflammatory signaling is dysregulated in IPF. Exo inhalation treatments downregulated several pathways involved in TGF‐β signaling, while inhaled miR‐30a demonstrated select gene downregulation. Because exosomes are comprised of various molecular cargo, additional components or their combinations may broaden signaling interactions, and regulation through phosphorylation, and alter gene activation related to TGF‐β.

The miR‐30a and Exo may interact in the lung to both reduce alveolar epithelial cell loss and induce myofibroblast de‐differentiation.^[^ ^34^ ^]^ Prior research showed apoptotic‐resistant fibroblasts are accumulated in IPF and inhibition of IAP proteins enhances myofibroblast apoptosis in a bleomycin model of IPF.^[^ ^35^ ^]^ Both miR‐30a and Exo treatments significantly reduced cIAP‐2 expression in pulmonary tissue. Tissue homeostasis after miR‐30a and Exo inhalation was promoted through the clearance of dysfunctional mitochondria indicated by the downregulation of CytoC,^[^ ^36^ ^]^ the promotion of myofibroblast and immune cell apoptosis through decreased BCL‐2 expression,^[^ ^37^ ^]^ and deactivation of fibroblasts indicated by the downregulation of BAX.^[^ ^38^ ^]^ IGF‐2, p21, and BID are upregulated in IPF and contribute to enhanced ECM deposition,^[^ ^39^ ^]^ suppressed alveolar regeneration,^[^ ^40^ ^]^ and alveolar epithelial cell apoptosis,^[^ ^41^ ^]^ respectively. Notably, miR‐30a and Exo treatments significantly reduced the expression of these profibrotic genes.

Both inhaled miR‐30a and Exo reduced proinflammatory cytokine signaling involved in profibrotic vascular modeling, fibroblast proliferation, inflammatory cell infiltration at the vascular endothelium, and the accumulation of leukocytes by MIP‐2,^[^ ^42^ ^[^ VCAM‐1,^[^ ^43^ ^[^ P‐Selectin,^[^ ^44^ ^]^ and L‐Selectin^[^ ^45^ ^]^ downregulation, respectively. Together, inhalation of miR‐30a and Exo promoted tissue homeostasis in bleomycin‐induced IPF by promoting myofibroblast de‐differentiation, which subsequently restored the alveolar epithelium while downregulating profibrotic vascular remodeling and proinflammatory cell recruitment.

The role of hsa‐miR‐30a‐3p is not well defined in pulmonary fibrosis. RNA quantification revealed significant downregulation of CNPY2 after inhaled miR‐30a treatments and protein analysis confirmed decreased CNPY2 expression, as well as downstream PERK/DDIT3 signaling.^[^ ^46^ ^]^ The miR‐30a‐3p was previously identified as a key regulator of the oncogene CNPY2 and inhibited epithelial‐mesenchymal transition (EMT) in human lung adenocarcinoma.^[^ ^47^ ^]^ Similarly, miR‐30a treatments suppressed EMT away from the α‐SMA^+^ myofibroblast signature by downregulating N‐cadherin relative to E‐cadherin. However, several recent single‐cell RNA sequencing studies of IPF lung samples have not identified changes in the CNPY2/PERK/DDIT3 pathway.^[^ ^48^, ^49^ ^]^ This may be due to limitations in capturing all relevant signaling pathways, particularly those that are context‐dependent.

Together, these data demonstrate that inhaled miR‐30a attenuated bleomycin‐induced pulmonary fibrosis by promoting myofibroblast de‐differentiation through the downregulation of the CNPY2/PERK/DDIT3 signaling pathway.

We acknowledge several limitations to this work. First, large‐scale production of both miR‐30a and Exo is limited by the liposome loading method of electroporation and the costly scale‐up requirements for exosome production. Lipid nanoparticle formulation for charge‐driven encapsulation of miRNA will need to be formulated and optimized for inhaled delivery in humans. In addition, further cell‐specific analysis of apoptotic markers, cytokines, and CNPY2/PERK/DDIT3 is necessary to better understand the specific pharmacodynamics of inhaled miR‐30a and Exo.

In summary, we developed antifibrotic drugs miR‐30a and Exo for the treatment of pulmonary fibrosis through dry powder inhalation. We determined that miRNA hsa‐miR‐30a‐3p plays a role in pulmonary function improvement and fibrotic regression by inducting EMT through de‐differentiation of pulmonary myofibroblasts.

## 4 Experimental Section

### Cell Culture

Human LSCs were generated from healthy human whole lung samples from the Cystic Fibrosis and Pulmonary Diseases Research and Treatment Center at the University of North Carolina at Chapel Hill and expanded as previously described.^[^ ^12^, ^50^, ^51^ ^]^ IPF patient‐derived fibroblast cells were purchased from Lonza. LSCs were cultured in Iscove's Modified Dulbecco's Medium with 20% fetal bovine serum. Lung fibroblast cell line HLF1 cells were purchased from the American Type Culture Collection (ATCC, CCL‐153) and cultured in Kaighn's Modification of Ham's F‐12 Medium with 10% fetal bovine serum. Media was changed every 2–3 days until the cells reached 70–80% confluence. Serum‐free secretome (LSC‐Secretome) was generated and collected as previously described.^[^ ^53^, ^54^ ^]^ All procedures performed in this study involving human samples were in accordance with the ethical standard of the institutional research committee and with the guidelines set by the Declaration of Helsinki.

### Nanoparticle Isolation

Exosomes secreted by LSCs were collected and isolated from LSC‐Secretome using an ultrafiltration method^[^ ^54^ ^]^ as previously described.^[^ ^17^, ^18^, ^55^ ^]^ Briefly, the filtered secretome was pipetted into a 100 kDa Amicon centrifugal filter unit (MilliporeSigma, Burlington, MA, USA) and centrifuged at 4000 rpm at 4 °C. After all media passed through the centrifugal filter unit, remaining exosomes were detached from the filter and resuspended using 1X Dulbecco's phosphate‐buffered saline (DPBS; ThermoFisher Scientific, Waltham, MA, USA) with 25 mm trehalose (MilliporeSigma, Burlington, MA, USA) for further analysis.

### Loading of miRNA into Liposomes

Pegylated Remote Loadable Liposomes were purchased from Avanti Polar Lipids (Avanti Polar Lipids, Inc, Alabaster, AL, USA). The hsa‐miR‐30a‐3p mimic (HMI0455; Sigma–Aldrich, St. Louis, MO, USA) was loaded into liposomes via electroporation^[^ ^56^ ^]^ as previously described,^[^ ^17^, ^18^, ^57^ ^]^ generating miR‐30a. Briefly, one billion nanoparticles from each sample were diluted in Gene Pulser Electroporation Buffer (Bio‐Rad, Hercules, CA, USA) at a 1:9 ratio of nanoparticles to buffer. One hundred twelve microgram of hsa‐miR‐30a‐3p mimic was added to the nanoparticle‐buffer solution and transferred to an ice‐cold 0.4 cm Gene Pulser/MicroPulser Electroporation Cuvette (Bio‐Rad). The electroporation cuvette was inserted into the Gene Pulser Xcell Total System (Bio‐Rad) and electroporated under the following conditions: pulse type: square waveforms; voltage: 200 V; pulse length: 10 msec; number of pulses: 5; pulse interval: 1 s. The electroporation buffer was filtered out of the loaded nanoparticles by the ultrafiltration method above. In this study, the loading efficiency is calculated as 56%.

### Characterization of Nanoparticles

The morphology of NPs was confirmed through TEM imaging (JEOL JEM‐2000FX, Peabody, MA, USA). The NPs were fixed with 4% paraformaldehyde (PFA; Electron Microscopy Sciences, Hatfield, PA, USA) and 1% glutaraldehyde (Sigma‐Aldrich, St. Louis, MO, USA) onto 100 mesh copper grids (Electron Microscopy Sciences, Hartfield, PA, USA) and stained with Vanadium Negative Stain (ab172780; Abcam, Cambridge, United Kingdom). The NP concentrations and mean diameters were quantified by NTA (NanoSight NS3000, Malvern Panalytical, Malvern, UK).

### SDS‐PAGE and Western Blot

NPs were characterized and protein expression in lung lysate was evaluated by immunoblotting as previously described.^[^ ^17^, ^18^ ^]^ The membranes were blotted against anti‐CD63 (PA5‐100713; ThermoFisher Scientific, Waltham, MA, USA), anti‐CD9 (PA5‐85955; ThermoFisher Scientific, Waltham, MA, USA), anti‐CNPY2 (PA5‐100135; ThermoFisher Scientific, Waltham, MA, USA), anti‐E‐cadherin (ab231303; Abcam, Cambridge, United Kingdom), anti‐N‐cadherin (ab76011; Abcam, Cambridge, United Kingdom), anti‐PERK (ab229912; Abcam, Cambridge, United Kingdom), anti‐DDIT3 (ab11419; Abcam, Cambridge, United Kingdom), and anti‐β‐actin (MA5‐15739; ThermoFisher Scientific, Waltham, MA, USA) primary antibodies at 4 °C for 24 h for lung lysate samples and 1 week for NP lysate samples. After incubation, the membranes were incubated with the corresponding goat anti‐rabbit (ab6721; Abcam, Cambridge, United Kingdom) and goat anti‐mouse (ab6789; Abcam, Cambridge, United Kingdom) HRP‐conjugated secondary antibodies. Band intensities were analyzed using ImageJ analysis software (NIH; https://imagej.nih.gov/ij/).

### qPCR

MiRNA was isolated using the miRNEasy kit (QIAGEN, Hilden, Germany) per the manufacturer's instruction. The concentration and purity of RNA were measured using the NanoDrop One (ThermoFisher Scientific, Waltham, MA, USA). Reverse transcription (RT) reactions were performed using the TaqMan MicroRNA Reverse Transcription Kit (ThermoFisher Scientific, Waltham, MA, USA) to generate cDNA per the manufacturer's instruction. The TaqMan™ Universal PCR Master Mix, no AmpErase UNG (ThermoFisher Scientific, Waltham, MA, USA) was used for qPCR on the qTOWER3 real‐time PCR thermal cycler (Analytik Jena, Jena, Germany). In vitro qPCR experiments used 20 ng of RNA from LSCs co‐cultured with NPs at a concentration of 1 billion particles/9.6 cm^2^ after 48 h. In vivo qPCR experiments used 10 ng of RNA from the mouse whole middle lung lobe that was dissociated using the gentleMACS Dissociator (Miltenyi Biotec, Auburn, CA, USA), gentleMACS M tubes (Miltenyi Biotec, Auburn, CA, USA), and 1 mL QIAzol lysis reagent (QIAGEN, Hilden, Germany). The primers used for RT and qPCR are shown in Table S1 (Supporting Information).

### Animal Procedures

Six‐week‐old male CD1 mice (022) were obtained from Charles River Laboratory (Wilmington, MA, USA). Pulmonary fibrosis was induced through intratracheal instillation of bleomycin sulfate solution (203 401; MilliporeSigma, Burlington, MA, USA) at a 3 U kg^−1^ dose. NP treatments began 10 days after bleomycin insult to simulate chronic pulmonary fibrosis. The mice were treated with seven consecutive treatments of miR30a, Exo, or PBS (Sham and IPF) through DPI. Mice who received miR30a were dosed at 1 µg hsa‐miR‐30a‐3p per treatment. Mice who received Exo were dosed at 1e9 ± 4.1e8 particles. Post 24 h of the final treatment, the mice were sacrificed and their organs were excised. Blood was collected in Vacuette ethylenediaminetetraacetic acid (EDTA) tubes (Greiner Bio‐One, Kremsmünster, Austria) and centrifuged at maximum speed for 5 min to separate serum. All animal studies complied with the requirements of the Institutional Animal Care and Use Committee (IACUC) of North Carolina State University (protocol number 22–431).

### Dry Powder Inhaler Fabrication and Powder Formulation

A dry powder inhaler for nanoparticle inhalation to mice was fabricated and modified as previously described.^[^ ^18^, ^58^ ^]^ NPs were formulated for dry powder inhalation as previously described.^[^ ^18^ ^]^ Briefly, the nanoparticles were formulated into dry powder through lyophilization (Labconco, Kansas City, MO, USA). The nanoparticles were diluted in DPBS with 25 mm trehalose at a 1:8 ratio of nanoparticle solution volume to DPBS with 25 mm trehalose solution volume. Trehalose solution serves as a cryoprotectant and bulking agent for sufficient powder production. Diluted nanoparticle solutions were stored at −80 °C overnight and lyophilized for 24 h.

### Pulmonary Function Tests

Pulmonary function tests included in this manuscript are resistance, elastance, hysteresis area, compliance, inspiratory capacity, and forced expiratory volume to FVC (FEV0.2/FVC). Baseline PFTs were taken in healthy mice on the flexiVent (SCIREQ Inc., Montreal, Canada). Endpoint PFTs were taken 24 h after the final DPI treatment. The mice were anesthetized with an intraperitoneal injection of ketamine and xylazine solution (1:1) and intubated with a 20‐gauge cannula. PFTs were repeated in triplicates and nonexcluded averages generated baseline and endpoint values. Normalized PFTs divided endpoint values by baseline values for each individual mouse.

### Histology

H&E, Gomori's trichrome, and sirius red stains were performed on paraffin‐embedded tissue sections and imaged by the Leica DMi8 microscope (Leica Microsystems, Wetzlar, Germany). H&E images were used for Ashcroft scoring.^[^ ^59^ ^]^ Ashcroft scores were generated by averaging the scores of four representative images per mouse from three blinded scorers. Immunostaining was performed on frozen tissue slides as previously described.^[^ ^17^, ^18^, ^52^ ^]^ Briefly, immunostaining was performed on tissue slides fixed in 4% paraformaldehyde (PFA) (Electron Microscopy Sciences; 15 710) followed by permeabilization and blocking with Dako Protein blocking solution (DAKO; X0909) containing 0.1% saponin (Sigma–Aldrich; 47 036) prior to antibody staining. Slides were stained at a dilution of 1:200 with primary and secondary antibodies. Tissues were immunolabeled with anti‐Aqp5 (ab78486; Abcam, Cambridge, United Kingdom), anti‐ProSPC (ab211326; Abcam, Cambridge, United Kingdom), anti‐vWF (ab287962; Abcam, Cambridge, United Kingdom), and anti‐α‐SMA (ab5694; Abcam, Cambridge, United Kingdom) primary antibodies diluted in Dako Protein blocking solution and its corresponding goat anti‐rabbit (ab150077; Abcam, Cambridge, United Kingdom) AF 488‐conjugated secondary antibody diluted in Dako Protein blocking solution. Slides were mounted with ProLong Gold Antifade Mountant (Invitrogen, Waltham, MA, USA) and ProLong Gold Antifade Mountant with DAPI (Invitrogen, Waltham, MA, USA). Slides were imaged on the Olympus FLUOVIEW confocal laser scanning microscope (Olympus; FV3000, Shinjuku, Tokyo, Japan) with an Olympus UPlanSAPO 10x objective (Olympus; 1‐U2B824, Shinjuku, Tokyo, Japan). Tissue slides were analyzed using ImageJ analysis software. All images were acquired by blinded individuals.

### Hydroxyproline Assay

Hydroxyproline concentrations were determined using lyophilized lung tissue per the manufacturer's instruction (MAK008; Sigma‐Aldrich, St. Louis, MO, USA). Briefly, 10 mg tissue per sample was hydrolyzed at 120 °C for 3 h. Hundred microliters of the Chloramine T/Oxidation Buffer mixture was added to each sample and incubated at room temperature for 5 min. Hundred microliters of the Diluted DMAB reagent was added to each sample and incubated for 90 min at 60 °C. Then, absorbance at 560 nm was measured.

### ELISAs and Arrays

The liver function was evaluated by serum ALT (ab282882; Abcam, Cambridge, United Kingdom) and serum AST (ab263882; Abcam, Cambridge, United Kingdom) levels by sandwich‐based enzyme‐linked immunosorbent assays (ELISAs) per manufacturer's instruction. TGF‐β signaling was analyzed by high‐throughput ELISA from murine lung tissue lysate per the manufacturer's instruction (PTG176; Full Moon BioSystems, Sunnyvale, CA, USA). The top target genes of hsa‐miR‐30a‐3p were determined using the TargetGene 8.0 database.^[^ ^60^ ^]^ The top 46 genes were analyzed using a customized plate (ScienCell, Carlsbad, CA, USA) and corresponding GoldNStart TaqGreen qPCR Master Mix (MB6018; ScienCell, Carlsba, CA, USA) for qPCR analysis. Its corresponding volcano plot was created using RStudio (RStudio, Boston, MA, USA). Cytokines (AAM‐CYT‐1000‐8; RayBiotech, Norcross, GA, USA) from murine serum were evaluated by sandwich‐based ELISA per the manufacturer's instruction. Apoptotic markers (AAM‐APO‐1‐8; RayBiotech, Norcross, GA, USA) from murine lung tissue lysate were evaluated by sandwich‐based ELISA per the manufacturer's instruction.

### Duel‐Luciferase Reporter Assay

The CNPY2 3′UTR, containing the predicted miR‐30a target site (AAAAGTCA), was cloned into the pGL3‐Promoter vector (Promega, E1761), positioned downstream of the TAA sequence and upstream of the polyadenylation signal. The constructs pGL3‐Promoter‐miR‐30a, along with the miR‐30a mimic and the pRL‐TK renilla luciferase control reporter vectors (Promega, E2241), were co‐transfected into lung fibroblast cells. The Dual‐Luciferase Reporter Assay System (Promega, E1910) was then employed to conduct the dual‐luciferase reporter assay, following the manufacturer's instructions.

### Statistical Analysis

Statistical analysis was performed using GraphPad Prism analysis software (GraphPad Software Inc., San Diego, CA, USA). Results are shown as the mean ± standard deviation. Comparisons among the two groups were performed using an unpaired *t*‐test, followed by Welch's correction test. Comparisons among more than two groups were performed using a parametric one‐way ANOVA test or two‐way ANOVA, followed by Bonferroni's multiple comparisons test. *p* ≤ 0.05 was considered statistically significant. The legend is as follows: ^*^ *p*‐values ≤ 0.05; ^**^ *p*‐values ≤ 0.01; ^***^ *p*‐values ≤ 0.001; ^****^ *p*‐values ≤ 0.0001; *ns* = not significant.

## Conflict of Interest

The authors report no conflict of interest.

## Supporting information



Supporting Information

## Data Availability

The data that support the findings of this study are available from the corresponding author upon reasonable request.
