# Supplementary material for: Inhalable Hsa‐miR‐30a‐3p Liposomes Attenuate Pulmonary Fibrosis
Source: Adv Sci (Weinh). 2025 Mar 22;12(19):2405434. doi: 10.1002/advs.202405434 (PMC12097057; doi:10.1002/advs.202405434)
Supplement: Supplementary file 1 — Supporting Information [file ADVS-12-2405434-s001.docx]

Supporting Information

Inhalable hsa-miR-30a-3p Liposomes Attenuate Pulmonary Fibrosis

Shuo Liu, Kristen D. Popowski, Christina M. Eckhardt, Weihang Zhang, Junlang Li, Yujia Jing, Dylan Silkstone, Elizabeth Belcher, Megan Cislo, Shiqi Hu, Halle Lutz, Asma Ghodsi, Mengrui Liu, Phuong-Uyen C. Dinh* & Ke Cheng*

**
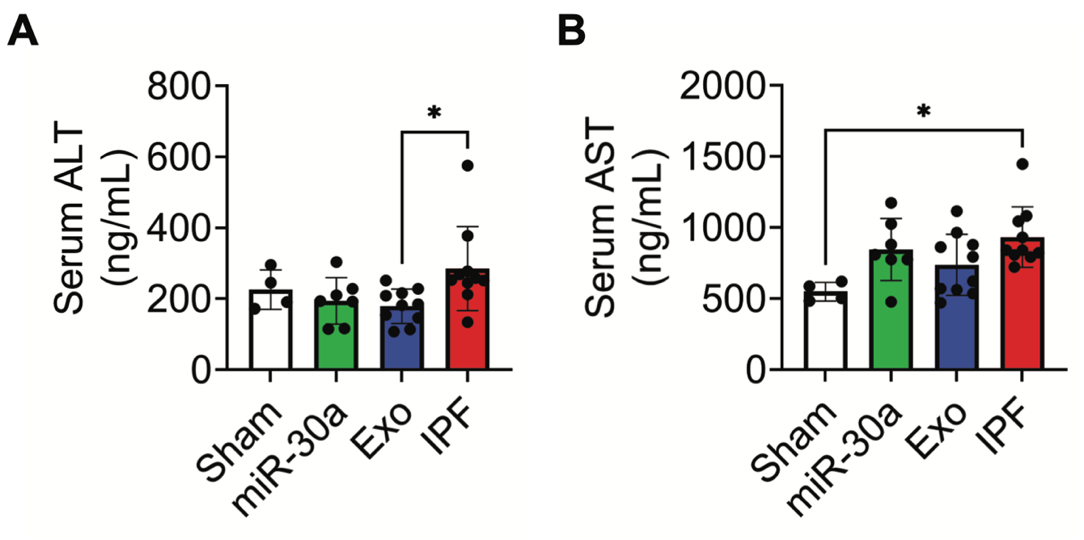
**

**Figure S1.** Evaluation of liver function following DPI treatments. A) Quantification of ALT levels from mouse serum by ELISA; n=4-10. B) Quantification of AST levels from mouse serum by ELISA; n=4-10. *P* values were determined by one-way ANOVA using GraphPad PRISM software. **p* < 0.05, ** *p* < 0.01, *** *p* <0.001, **** *p* < 0.0001; *ns*, not significant.


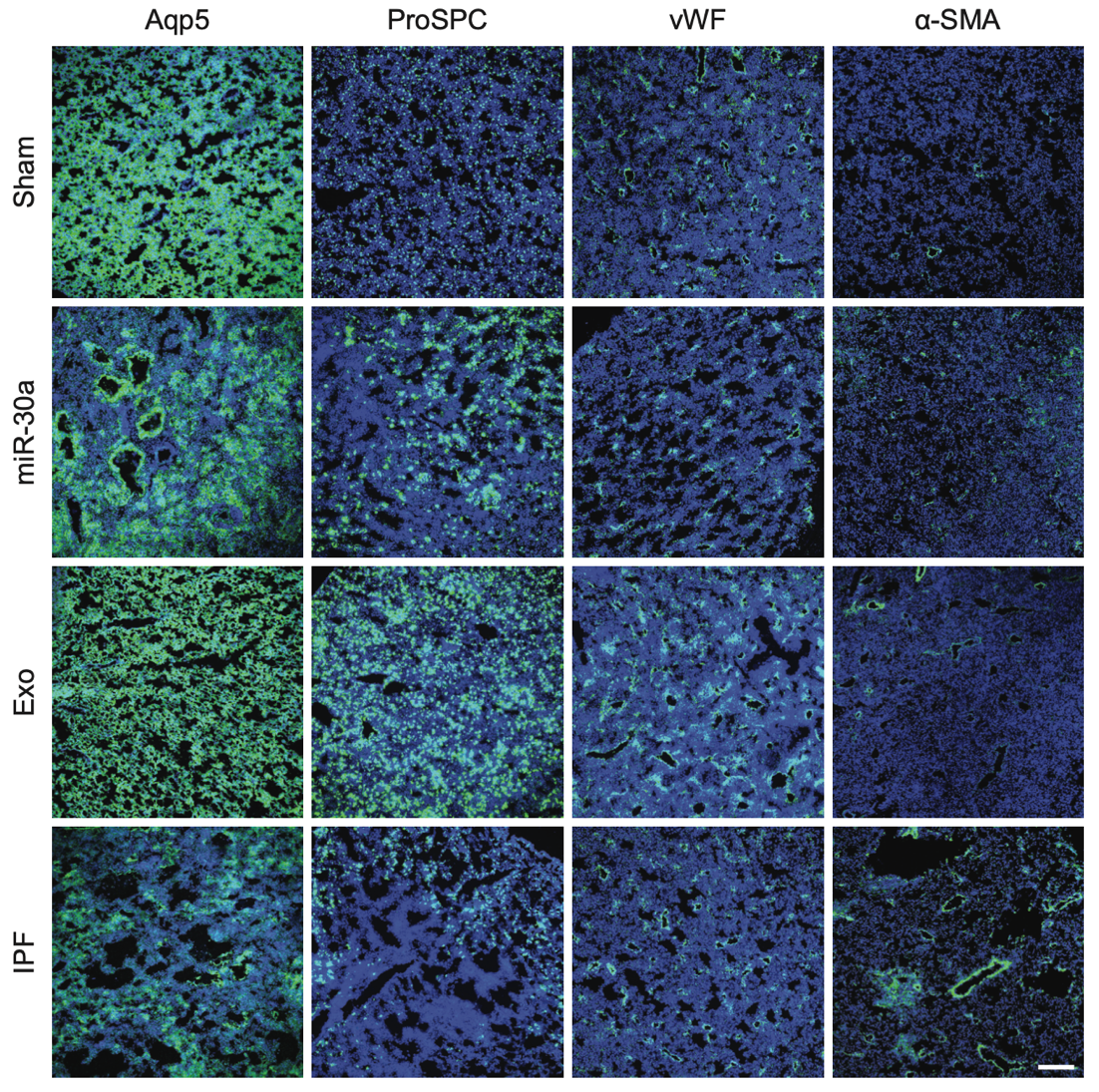


**Figure S2.** Immunostaining of mouse lung tissues. Representative immunostaining images of lung tissue sections from mice that received miR-30a, Exo, or PBS (Sham and IPF). Sections were stained for Aqp5, ProSPC, vWF, or α-SMA (green) and DAPI (blue). scale bar = 188 μm.

**Figure S3.** Pulmonary function changes following pirfenidone administration. A) Quantification of endpoint resistance normalized to baseline resistance per mouse; n=5 per group. B) Quantification of endpoint compliance normalized to baseline compliance per mouse; n=5 per group. C) Quantification of endpoint elastance normalized to baseline elastance per mouse; n=5 per group. D) Quantification of endpoint inspiratory capacity normalized to baseline inspiratory capacity per mouse; n=5 per group. E) Quantification of endpoint hysteresis area normalized to baseline hysteresis area per mouse; n=5 per group. *P* values were determined by T test using GraphPad PRISM software. * *p* < 0.05, ** *p* < 0.01, *** *p* <0.001, **** *p* < 0.0001; *ns*, not significant.

**Figure S4.** Evaluation of lung fibrosis serum biomarkers. A) Quantification of serum MMP levels from mouse serum by ELISA; n=4, normalized to sham. B) Quantification of serum MUC1 levels from mouse serum by ELISA; n=4, normalized to sham. *P* values were determined by one-way ANOVA using GraphPad PRISM software. **p* < 0.05, ** *p* < 0.01, *** *p* <0.001, **** *p* < 0.0001; *ns*, not significant.

**Figure S5.** Antifibrotic effects of has-miR-30a-3p in IPF patient-derived fibroblasts. A) Immunoblots of CNPY2, α-SMA, and β-Αctin in human IPF fibroblasts primary cells with has-miR-30a-3p administration. B) Immunoblots of E-cadherin and β-actin in human IPF fibroblasts primary cells with has-miR-30a-3p administration.

**Figure S6.** Original blotting of western blot results for Figure 1b.

**
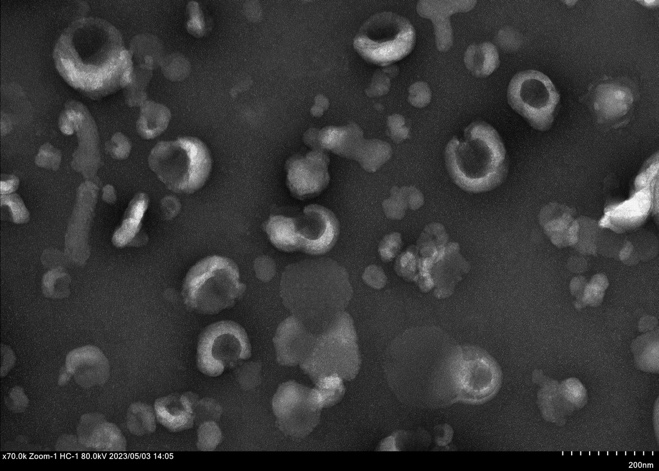
**
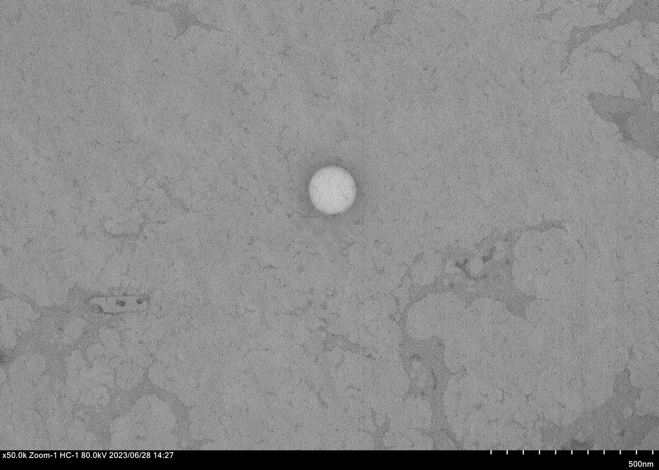


**Figure S7.** Full images of Figure 1C.

**Figure S8.** Original blotting of western blot results for Figure 4E.

**Figure S9.** Original blotting of western blot results for Figure 5C. The lane number represents the order of the figures from left to right.

**Figure S10.** Original blotting of western blot results for Figure S5A. The lane number represents the order of the figures from left to right.

**Figure S11.** Original blotting of western blot results for Figure S5B. The lane number represents the order of the figures from left to right.

| **Name** | **Sequence** |
| --- | --- |
| hsa-miR-30a-3p | 000416, ThermoFisher Scientific |
| Rnu6 | NR_002752, ThermoFisher Scientific |
| CNPY2-F | AGGGCTCTGGTGGATGAATTA |
| CNPY2-R | CTCTGAGCGGGCATAAGGT |
| ACTA2-F | GTGTTGCCCCTGAAGAGCAT |
| ACTA2-R | GCTGGGACATTGAAAGTCTCA |
| COL1A1-F | GAGGGCCAAGACGAAGACATC |
| COL1A1-R | CAGATCACGTCATCGCACAAC |
| DDIT3-F | GGAAACAGAGTGGTCATTCCC |
| DDIT3-R | CTGCTTGAGCCGTTCATTCTC |

**Table S1. The primers used in PCR.**
